# The Physiological Action of Commercial Aconitines

**Published:** 1882-06

**Authors:** 


### THE PHYSIOLOGICAL ACTION OF COMMERCIAL ACONITINES.

The physiological action of different commercial aconitines forms the subject of a paper by Prof. P. C. Plugge, of Groningen (Netherlands), published in Virchow's *Archiv*, vol. lxxxvii., p. 410,* from which we abstract the following:

During the experiments conducted by Prof. Plugge, in conjunction with Prof. Huisinga, to ascertain the fatal dose of certain aconitines submitted to them (in consequence of the poisoning case at Winschoten, see *New Remedies*, 1880, p. 293), Prof. Plugge thought he could recognize qualitative differences alongside of the quantitative effects; but the object of the investigation being mainly the discovery of the latter, and the quantities of the materials at disposal being limited, no special attention could be paid to the former.

For the purpose of the present investigation, the following seven different kinds of aconitine were employed:

1. *Nitrate of aconitine*, from Petit—in form of hard white crystals.
2. *Nitrate of aconitine*, from Morson—a yellowish-brown powder. This was a "pseudaconitine" [that is the alkaloid extracted from the Himalaya aconite (*Aconitum ferox*, Wallich).]†
3. *Nitrate of aconitine*, from Hottot—a white powder.
4. *Nitrate of aconitine*, from Hopkins and Williams, a transparent gum-like mass of greenish-brown color (perhaps a pseudaconitine?).
5. *Nitrate of aconitine*, from Merck—a yellowish light-brown powder.

---

* Communicated by the author.

† If the term pseudaconitine, in the original, is used in the sense in which it is now generally understood. The words in brackets are added by us.—Ed. N. R.

6. *Sulphate of aconitine*—a grayish-white powder.

7. *Nitrate of aconitine*, from Trommsdorff—a grayish-white gum-like mass. (This was obtained from Friedländer, of Berlin, but was made by Trommsdorff.)

The above-named kinds of aconitine were used in aqueous, one-half or one per cent solution upon frogs ; and while it was not the special object of the author to determine the fatal dose of each sample, yet his results justified him to classify the samples, according to their comparative efficacy, in the succession given above. The most active is the French, of Petit, which is even stronger than Morson's, and the weakest is Trommsdorff's.

Want of space and the purely physiological interest attached to the experiments do not justify us in presenting the details of the experiments contained in the author's paper ; but the results may be summed up as follows :

1. Though the different kinds of aconitine exhibit a difference in the quantity of effect no such difference could be detected in the quality of the same, all of them (even pseudaconitine) affecting the heart, respiration, motor nerves, etc., in precisely the same manner.

2. Aconitine and pseudaconitine paralyze the peripheric, intramuscular terminations of the motor nerves, resembling, in this respect, curare.

3. The nerve trunk is not paralyzed by these alkaloids.

4. The sensory nerves are not at all, or, at most, very faintly, paralyzed.

5. Where general paralysis apparently supervenes, this appears to be only due to the effect upon the peripheric nerve-ends.

6. The muscles retain their excitability, even after doses of aconitine which are five to ten times larger than those which paralyzed the nerve-ends.

The following observations are also recorded as having been incidentally noticed :

7. The pupil is frequently dilated, but not always.

8. Respiration soon becomes laborious, and ceases entirely after a few minutes. The phenomenon is quite constant.

9. A slimy secretion of the skin (in frogs) was frequently noticed. Its quantity was different, according to the kind of aconitine employed.

10. Yawning and retching occur almost always (both in *Rana esculenta* and in *Rana temporaria*).

11. The blood of the poisoned animal generally has a dark-violet color, so that the full veins appear black. Only Schuchart's and Trommsdorff's preparations produce a much less intense change of color.

12. The heart generally stops in diastole, densely filled with dark violet-red blood ; the ventricles beat more strongly than the auricles. The poisoned but still pulsating heart cannot be made motionless either by excitement of the pneumogastric nerve or by that of the sinus. Just after ceasing to beat, it may be brought in motion again by galvanic excitement.—*New Remedies.*

